# Achieving Durable Forehead Line Outcomes With DaxibotulinumtoxinA: Early Experience in Real-World Clinical Practice

**DOI:** 10.1093/asjof/ojae023

**Published:** 2024-04-16

**Authors:** Michael Lafkas

## Abstract

**Background:**

Irrespective of botulinum toxin type A (BoNT-A) product used, differences in duration between the frontalis and glabella can be a challenge. Since the approval of daxibotulinumtoxinA for injection (DAXI, DAXXIFY; Revance, Nashville, TN), injectors have been eager not only to reproduce the durable results observed in the glabella but also to achieve an extended duration in the frontalis.

**Objectives:**

To describe how the author has successfully modified their injection technique to achieve extended DAXI duration in the frontalis.

**Methods:**

In this study, the author presents an approach to obtaining longevity in the forehead with DAXI based on clinical experience performing more than 400 treatments on more than 250 patients with DAXI since product approval.

**Results:**

DAXI has a limited diffusion profile. If patients are not injected in the mid-to-low frontalis directly, compensatory motion of the lower frontalis leads to premature return of movement, which can result in suboptimal DAXI injection and shortened patient-reported duration. With previous generations of BoNT-A products, some degree of migration from the upper frontalis injection sites and/or the glabella injection sites into the mid and low frontalis is sufficient to prevent this effect, but the precision of DAXI demands that these fibers be more deliberately addressed with a larger number of individual injections.

**Conclusions:**

Through the use of more injection points, a wider area of treatment, and customization to fit patient needs, duration similar to that observed in clinical studies (20.9 weeks) can be achieved using between 20% and 33% fewer units of DAXI.

**Level of Evidence: 4:**

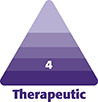

In aesthetic medicine, strict on-label use of devices is relatively uncommon. Rather, devices are adapted to treat multiple areas of the face and body in order to better meet patient needs. Thus, data from pivotal trials are often viewed as establishing a baseline understanding of efficacy, tolerability, safety, and important patient-reported outcomes like satisfaction, but not necessarily reflecting real-world product use. For example, botulinum toxin type A (BoNT-A) products for aesthetic use are most commonly tested for the correction of glabellar lines; however, BoNT-A is regularly used to treat a wide range of muscles in the face and body, including the frontalis, orbicularis oculi, platysma, and calf muscles, among many others.^1-3^

When treating the upper face, the injector must pay careful attention to interactions between muscle groups, and it is rare that the glabella is treated in isolation without also addressing forehead lines and/or lateral canthal lines. Although daxibotulinumtoxinA for injection (DAXI, DAXXIFY; Revance, Nashville, TN) is FDA cleared to treat glabellar lines, many injectors are keen not only to reproduce the durable results observed across a number of clinical trials in the glabella but also to achieve an extended duration in the frontalis.^4^ Irrespective of the BoNT-A product used, a difference in duration between the frontalis and glabella is a challenge in real-world clinical practice. Because of differences in muscle dynamics and activity level, as well as the need to preserve natural-looking motion, patients may complain that the duration of effect in the frontalis is shorter than in the glabella, even when excellent injection technique has been used. For DAXI, especially when patients are paying a premium for durability, satisfaction hinges on long-lasting results in both the glabella and the frontalis.

In this study, the author presents an approach to obtaining longevity in the forehead with DAXI based on clinical experience performing more than 400 treatments on more than 250 patients with DAXI since product approval. The injection techniques described here are specific to DAXI and are based on the observation that DAXI does not appear to diffuse as far from the site of injection as other BoNT-A products. This clinical behavior is supported by preclinical data. When injected into the mouse gastrocnemius muscles, DAXI demonstrated significantly less inhibition of the adjacent tibialis anterior compared with onabotulinumtoxinA (ONA; Botox; Allergan Aesthetics, Dublin, Ireland), indicating less diffusion for DAXI.^5^ Furthermore, lower rates of dysphagia observed in DAXI clinical studies for cervical dystonia are suggestive of limited diffusion (4.7% for DAXI compared with 10%-25% for other BoNT-A products).^6-9^ Although the exact mechanism underpinning this divergence is unknown, the increased affinity of the DAXI excipient, which is positively charged at physiologic pH, for negatively charged nerve terminals may contribute.^10,11^

Published clinical data support a median time to loss of “none” or “mild” forehead line severity of up to 20.9 weeks for DAXI (32 U injected as 4 equal 8 U injections in the frontalis); however, in the author's clinic, similar duration can be achieved using between 20% and 33% fewer units of DAXI through the use of more injection points, a wider area of treatment, and customization to fit patient needs.^12^ Recommendations presented below for improving the longevity of the DAXI treatment effect in the frontalis are based on clinical experience and are not intended to assert any specific duration of effect, but rather are intended to shorten the learning curve for all aesthetic injectors so that patients can be better served.

## METHODS

Between December 2022 and April 30, 2023, the author treated over 250 patients in his practice with DAXI. The techniques presented here are based on personal experience and patient experience within a single practice.

The techniques presented here were developed during the course of clinical practice and were not part of a study. Further, there are no prospective or retrospective data to report; thus, IRB approval is not needed. All patients provided consent for treatment, and those presented here provided written consent for the use of their photographs. All patients were treated according to the guiding principles outlined in the Declaration of Helsinki.

## RESULTS

**Figure 1.**
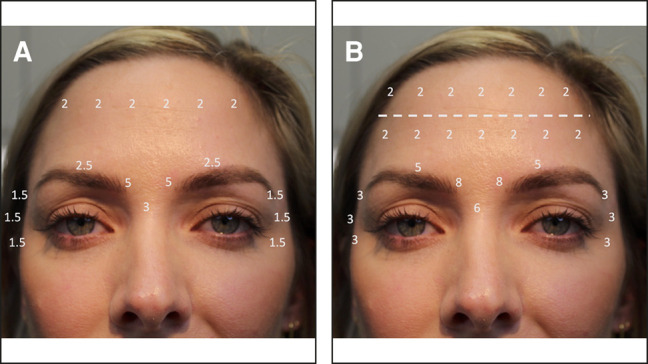
Injection points for onabotulinumtoxinA (A) and daxibotul inumtoxinA (Revance Therapeutics, Inc., Nashville, TN) (B). The numbers represent the number of units administered at each site for the respective products.

Although there are no “standard” injection patterns for BoNT-A in clinical practice, especially in the frontalis, the patient case shown in Figure 1 can serve as an example of how injection technique can be adapted to account for the unique features and behaviors of the DAXI formulation. A video describing the adaptation of this patient's injections to accommodate the unique diffusion properties of DAXI is also provided (Video). In each of the cases presented here, the number of units administered is based on reconstitution of a 100 U vial of DAXI with 1.25 cc of bacteriostatic saline and reconstitution of a 100 U vial of ONA with 2.5 cc of bacteriostatic saline. These were the reconstitution volumes used for each of the cases presented in this study.

**Figure 2.**
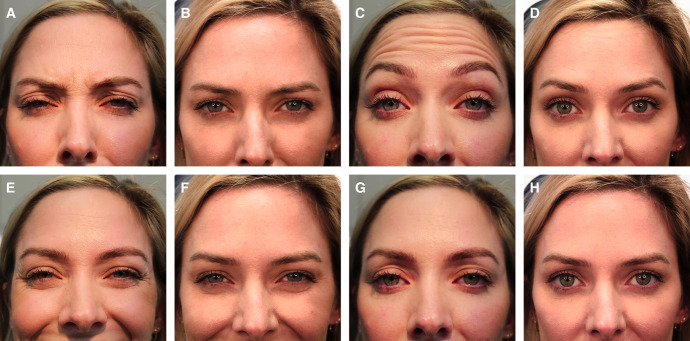
Case patient 1. A 32-year-old female patient 3 months and 9 days after injection with 39 total units of onabotulinumtoxinA (A, C, E, G). The patient is shown 4 weeks after injection with 78 total units of daxibotulinumto xinA (Revance Therapeutics, Inc., Nashville, TN; B, D, F, H).

**Case 1.** In Figure 2, a case study patient is shown 3 months and 9 days following injection with 39 total units of ONA (Figure 2A, C, E, G), when she presented for retreatment with DAXI (Revance Therapeutics, Inc., Nashville, TN), and 1 month following injection with 78 total units of DAXI (Figure 2B, D, F, H). The injection patterns used for this individual are shown in Figure 1. Initial treatment with ONA consisted of 18 U in the glabella, 12 U in the frontalis, and 9 U in the lateral canthal lines. Treatment with DAXI consisted of 32 U in the glabella, 28 U in the frontalis, and 18 U DAXI in the lateral canthal lines.

**Figure 3.**
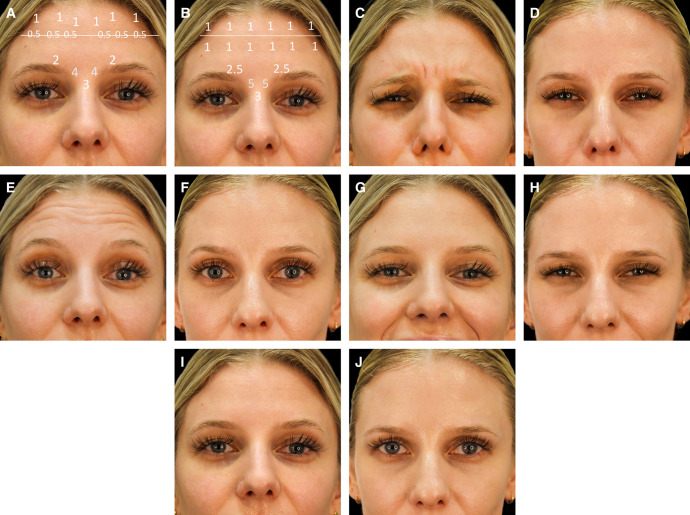
Case patient 2. A 27-year-old female patient 5 months and 27 days after injection with 24 total units of onabotulinumtoxinA (ONA), at which time she was retreated with 30 total units of daxibotulinumtoxinA (DAXI; Revance Therapeutics, Inc., Nashville, TN). Injection points for ONA and DAXI are shown in Panels A and B, respectively. The patient is shown prior to injection with DAXI (C, E, G, I) and 4 weeks after injection with 78 total units of DAXI (D, F, H, J).

**Figure 4.**
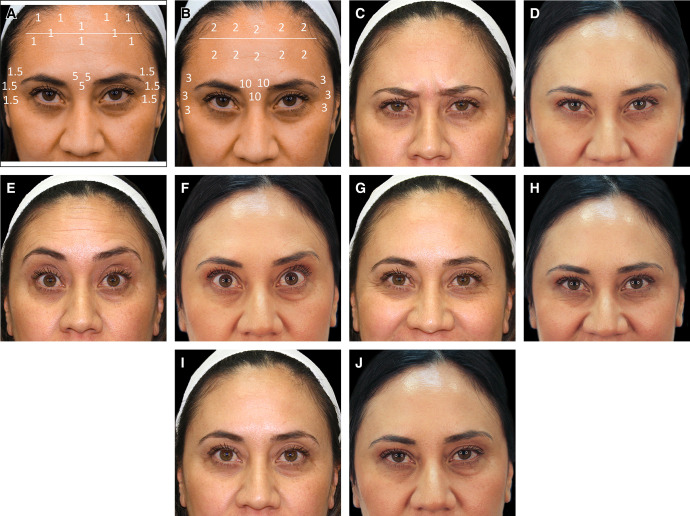
Case patient 3. A 45-year-old female patient 3 months and 9 days after injection with 34 total units of onabotulinumtoxinA (ONA) at which time she was retreated with 68 total units of daxibotulinumtoxinA (DAXI; Revance Therapeutics, Inc., Nashville, TN). Injection points for ONA and DAXI are shown in Panels A and B, respectively. The patient is shown prior to injection with DAXI (C, E, G, I) and 4 weeks after injection with 68 total units of DAXI (D, F, H, J).

**Case 2.** In Figure 3, a 27-year-old patient is shown 5 months and 27 days following injection with 24 total units of ONA, when she presented for retreatment with DAXI (Revance Therapeutics, Inc., Nashville, TN),. Injection points for ONA and DAXI are shown in Figure 3A and B, respectively. The patient is shown prior to injection with DAXI (Figure 3C, E, G, I) and 4 weeks after injection with 30 total units of DAXI in Figure 3D, F, H, and J. Initial treatment with ONA consisted of 15 U in the glabella and 9 U in the frontalis. Treatment with DAXI consisted of 18 U in the glabella and 12 U in the frontalis. The patient is shown at rest in Panels I and J.

**Case 3.** In Figure 4, a 45-year-old female patient is shown 10 months 2 days after injection with 34 total units of ONA, when she presented for retreatment with DAXI (Revance Therapeutics, Inc., Nashville, TN). The injection patterns for ONA and DAXI are shown in Figure 3A and B, respectively. The patient is shown at baseline, just prior to injection with DAXI in Figure 4C, E, G, and I and 4 weeks after injection with 68 total units of DAXI (Figure 4D, F, H, J). Initial treatment with ONA consisted of 15 U in the glabella, 10 U in the frontalis, and 9 U in the lateral canthal lines. Treatment with DAXI consisted of 30 U in the glabella, 20 U in the frontalis, and 18 U DAXI in the lateral canthal lines. The patient is shown at rest in Panels I and J.

## DISCUSSION

Although injections must be tailored to individual patient needs, the following guidance can be used to modify existing techniques to achieve better outcomes with DAXI.

### Dose

1. In general, the total dose needed in the frontalis is between 2 and 2.4 U of DAXI for every 1 U of ONA.
2. Treatment must be customized based on the individual patient’s muscle mass and movement. For example, a male patient typically receives 36 to 44 U of DAXI in the glabella and 24 to 32 U in the frontalis, whereas a female patient receives 28 to 36 U in the glabella and 16 to 24 U in the frontalis.

### Injection Patterns

1. Treatment must be customized based on the individual patient’s musculature. The frontalis can vary widely between patients, and individual anatomy should be inferred from the patient's dynamic line patterns.^13^
2. Use a sufficient number of injection points to distribute DAXI evenly in both the horizontal and vertical planes of the frontalis. Clinically, DAXI is very precise, and too few injections can lead to uneven denervation with compensatory frontalis muscle activity in undertreated portions of the frontalis. Most often, 25% to 50% more injection points are needed for DAXI compared with ONA in the same patient, and twice as many injection sites are needed for DAXI compared with abobotulinumtoxinA (ABO; Dysport; Galderma, Lausanne, Switzerland), which is known to have even greater diffusion characteristics.
3. With DAXI, the lower and midline frontalis must be directly treated. Because of the diffusion properties of earlier BoNT-A products, injections in the lower frontalis above the brow bone traditionally have been discouraged due to the risk of brow ptosis. However, the activity of DAXI appears to be more focal, and in patients where the mid and low frontalis are *not* injected, compensatory motion of the lower frontalis leads to premature return of movement, which can result in suboptimal DAXI injection and shortened patient-reported duration. With ONA, some degree of migration from the upper frontalis injection sites and/or the glabella injection sites into the mid and low frontalis is sufficient to prevent this effect, but the precision of DAXI demands that these fibers be more deliberately addressed. For practices that primarily use ABO, which is normally injected even higher in the frontalis and allowed to migrate downward over the 7 to 10 days following injection, failure to adapt injection patterns to accommodate DAXI's precise action and field leads to an even higher risk of excessive denervation in the upper frontalis, strong compensatory movement in the lower frontalis, and the perception of treatment failure or shortened duration with DAXI.
4. For DAXI, injections are administered in a pattern like that shown in Figure 1, as 2 rows: one in the upper frontalis and the other below the horizontal line of convergence (C-line) on the forehead where bidirectional movement of the upper and lower forehead skin meet: the skin of the lower forehead moves cranially, while the skin of the upper forehead moves caudally.^14^

### Patient Follow-up

1. If a patient complains of uneven effects or early wearing-off, they should be evaluated at peak efficacy following their treatment to determine whether unevenness is present at this time. Activity at peak effect is suggestive of compensatory muscle hyperactivity rather than a true wearing-off. In this case, injection points should be modified accordingly and documented in the treatment record so that future DAXI treatments can be optimized.
2. It is also important to make sure patients are aware that a slow return to baseline, starting from the time of peak effect, is expected, so that they do not conflate motion with absence of effect.

### Safety

In the author's experience, there have been no unique side effects observed either for DAXI or for sequential treatment following prior injection with ONA. Anecdotally, patients may find the DAXI injection to be slightly more uncomfortable (eg, stinging); however, this occurs in a minority of patients (∼20%-25% of patients) and has not been a deterrent to retreatment. Patients treated with DAXI do not generally request a return to treatment with their previous toxin product. In the author's practice, 7 of 8 patients continue to be treated with DAXI. As with any BoNT-A treatment, reinjection when the effects of the prior toxin are still present has an additive effect that is important to consider, especially in the frontalis, where some motion must be preserved for a natural-looking outcome. Using the approach to injection pattern modification described here, in the 200 patients treated by the author at the time of this writing, there were 3 cases of mild brow ptosis that resolved by 4 to 6 weeks and no cases of lid ptosis reported.

Given the unique formulation of DAXI, it is reasonable to expect that a learning curve will be encountered when expanding product use into off-label areas. By injecting DAXI differently and taking into account its limited diffusion, the treatment effect in the frontalis may be extended, supporting a more natural-looking and satisfying outcome for patients. The observations and cases reported here are important starting points for achieving better outcomes for patients; however, they are based on the experience of a single practitioner. There is a need to more systematically measure the duration achievable with DAXI in the frontalis using the described methods. A randomized controlled study is needed to more objectively assess outcomes using this technique using validated scales and patient-reported outcomes. Furthermore, long-term data are needed to assess the duration of the effect over time.

## CONCLUSIONS

By modifying the injection technique for DAXI to include more points lower in the frontalis, the duration of DAXI can be improved in this area, leading to better and more consistent aesthetic outcomes.
